# Distributed Health Literacy in the Maternal Health Context in Vietnam

**DOI:** 10.3928/24748307-20190102-01

**Published:** 2019-02-05

**Authors:** Shannon McKinn, Duong Thuy Linh, Kirsty Foster, Kirsten McCaffery

## Abstract

**Background:**

Previous health literacy research has often focused on individual functional health literacy, ignoring the cultural contexts through which many people experience health care.

**Objective:**

We aimed to explore the nature of maternal health literacy among ethnic minority women in a low-resource setting in Vietnam.

**Methods:**

Using a qualitative approach, we conducted focus groups with 42 pregnant women, mothers, and grandmothers of children younger than age 5 years from the Thai and Hmong ethnic groups. Semi-structured interviews were conducted with key informants and thematic analysis was performed.

**Key Results:**

The findings of our thematic analysis aligned well with the concept of distributed health literacy. We found that ethnic minority women drew upon family and social networks of health literacy mediators to share knowledge and understanding, assess and evaluate information, communicate with health professionals, and support decision-making. Family members were also involved in making health decisions that had the potential to negatively affect women and children's health.

**Conclusions:**

Family members are an important source of information for ethnic minority women, and they influence decision-making. Relatives and husbands of pregnant women could be included in maternal health education programs to potentially strengthen the health literacy of the whole community. The distributed health literacy concept can be used to strengthen health promotion messages and to reduce the risk of negative health outcomes. **[*HLRP: Health Literacy Research and Practice*. 2019;3(1):e31–e42.]**

**Plain Language Summary:**

Distributed health literacy refers to how health literacy skills and practices are distributed through social networks. This concept applies well to the maternal health context in Vietnam. Older women are trusted sources of information, and family influences decision-making during pregnancy. Women's limited autonomy increases the importance of family involvement. Distributed health literacy could be used to strengthen health promotion messages.

Health literacy comprises factors that determine the motivation and ability of people to gain access to, understand, communicate about, and use information in ways that promote and maintain good health (Berkman, Davis, & McCormack, 2010; Nutbeam, 1998). Health literacy research has largely focused on quantitative measures of health literacy in the context of disease treatment and health care (Smith & Carroll, 2017), often focusing on functional health literacy skills such as reading and numerical comprehension (Baker, Williams, Parker, Gazmararian, & Nurss, 1999; Chew, Bradley, & Boyko, 2004; Davis et al., 1991; Parker, Baker, Williams, & Nurss, 1995). This individual function-focused approach does not fully reflect the way that people experience health care, manage health conditions, and make decisions about their health, as it obscures social and cultural context (de Wit et al., 2017). As such, there is increasing debate about the usefulness and meaningfulness of the function-focused approach across all population groups, and subsequent moves toward developing measurements that embody broader concepts of health literacy (Osborne, Batterham, Elsworth, Hawkins, & Buchbinder, 2013; Sørensen et al., 2013). The focus on individual functional health literacy may be particularly irrelevant in settings where less emphasis is placed on the individual, and collective social units/structures such as families, shared norms, and traditions have more influence.

Maternal health literacy broadens the definition of health literacy from promoting and maintaining good health in the individual to promoting and maintaining good health of both the mother and child (Renkert & Nutbeam, 2001). It is associated with children's nutritional (Johri et al., 2016) and vaccination status (Johri et al., 2015) and it has been posited that significant, rapid improvement in maternal health literacy is achievable with appropriate and targeted interventions (Mobley et al., 2014; Smith & Carroll, 2017), even when reading and other functional skills are weak (Smith & Carroll, 2017). Improved maternal health literacy can empower women to address factors that affect outcomes for both mothers and children (Lu & Halfon, 2003; Mobley et al., 2014; Smith & Carroll, 2017). To date, there has been limited research on health literacy generally, or maternal health literacy specifically, in low- and middle-income non-Western settings. The concept of distributed health literacy may provide insights into understanding and improving maternal health literacy in such contexts.

Edwards, Wood, Davies, and Edwards (2015) explored how health literacy is distributed through family and social networks of people living with chronic conditions. They found that people with long-term conditions drew on the health literacy skills of others (i.e., health literacy mediators) to seek, understand, and use health information, demonstrating the potential of distributed health literacy for supporting people to manage their conditions more effectively. Edwards et al. (2015) describe four broad areas of distributed health literacy, where they found “health literacy was utilized and a range of health literacy skills and practices that were distributed around an individual by members of their social network” (p. 1186–1187): (1) shared knowledge and understanding, (2) accessing and evaluating information, (3) supporting communication, and (4) supporting decision-making. Previously, this model has been applied to people living with chronic conditions generally, and to people living with type 2 diabetes (Abreu, Nunes, Taylor, & Silva, 2018). Although pregnancy is an altered physiological state and not a disease or condition, pregnancy is often a time when a woman's engagement with the health system and health information increases. Thus, the distributed health literacy model may be useful to help us understand more about maternal health literacy, and information and support needed during pregnancy.

Dien Bien Province (DBP) is a mountainous province of Vietnam, predominantly populated by ethnic minority groups (80% of population) (UNICEF Viet Nam, 2011) who experience poorer health and economic outcomes than the average Vietnamese person (Ministry of Planning and Investment, 2015). Maternal and child health outcomes in DBP are particularly poor (General Statistics Office of Vietnam, 2015; World Health Organization, 2015), especially given Vietnam's status as a “fast-track” country for achieving Millennium Development Goals (MDGs) 4 and 5 (i.e., on track in 2012 to meet MDG targets ahead of comparable countries) (Kuruvilla et al., 2014). A key strategy in Vietnam's success has been expanding access to health facilities and increasing the health workforce to provide access to essential services, including skilled birth attendance and emergency obstetric care (Ministry of Health Viet Nam et al., 2014). However, this success has not been shared equally. Ethnic minority women are less likely than the Kinh majority to access antenatal care (79.9% vs. 99.2%) or give birth with a skilled attendant present (70.7% vs. 99.4%) (Chuong et al., 2018), and ethnic minority status has been found to be a significant determinant of health service utilization (Goland, Hoa, & Malqvist, 2012). Improving quality of care and equitable access to care for ethnic minorities and other marginalized groups continues to be a significant challenge for the Vietnamese government (Ministry of Health Viet Nam, 2014).

Previous research has shown that health professionals in DBP generally perceive communication issues as being related mostly to patient factors, such as language barriers, education, and literacy levels (McKinn, Duong, Foster, & McCaffery, 2017a). Many ethnic minority women do not understand information provided by health workers about pregnancy and childbirth, with health professionals increasingly relying on providing written information over interpersonal communication (McKinn, Duong, Foster, & McCaffery, 2017b). Health literacy has not been measured on a population level in DBP, but it is reasonable to assume a low level of health literacy given lower levels of educational attainment (Lee, Tsai, Tsai, & Kuo, 2010; Paasche-Orlow, Parker, Gazmararian, Nielsen-Bohlman, & Rudd, 2005), and the absence of functional literacy and Vietnamese language skills, particularly among ethnic minority women (UNICEF Viet Nam, 2011).

This study used a qualitative method to examine the nature of maternal health literacy in DBP by exploring which formal and informal sources of health information ethnic minority women access and trust, and how women draw on these resources and their social and family networks to apply their understanding of health information and make health decisions.

## Methods

Methods for the overall study have been published elsewhere, with the study setting, recruitment, data collection, and analysis processes extensively described (McKinn et al., 2017b). Relevant modifications and details of additional participants are described below.

### Study Design

This study used a qualitative, focused ethnographic design (Knoblauch, 2005), and takes a pragmatist theoretical stance (Cornish & Gillespie, 2009). The focused ethnographic approach allowed us to center culture while containing our focus to specific research objectives, with the field of investigation determined by pre-existing problem-focused and context-specific research questions (Higginbottom, Pillay, & Boadu, 2013; Knoblauch, 2005; McElroy et al., 2011).

### Study Location

The study was conducted in October 2015 in the Tuan Giao District, DBP. This is a rural district, 80 km from the provincial capital and divided into 19 communes, with a total population of approximately 82,000. The study was conducted in five communes, selected in cooperation with the District Health Service.

### Recruitment

Ethnic minority women who were currently pregnant, or mothers or grandmothers of children younger than age 5 years were eligible to participate in focus groups and were recruited with the assistance of the Vietnamese Women's Union (VWU). Key informants were recruited with the assistance of the VWU and commune health station staff. All participant information and consent forms were provided to participants in Vietnamese or translated orally into local languages (Thai and Hmong) if required. All participants gave written or oral consent. All participants were compensated 100,000 Vietnamese dong ($4.45 in U.S. currency) for their time. Ethics approval was obtained through the University of Sydney Human Research Ethics Committee. The research plan was approved by the DBP Public Health Service, the Tuan Giao District Health Service, and the VWU.

### Participants

We conducted eight focus groups with 42 community participants in five villages. Seven focus groups were conducted with pregnant women and/or mothers of children younger than age 5 years, and one focus group was conducted with grandmothers. Key informant interviews were conducted with one village health worker and one village midwife (**Table 1**).

We purposely sampled for diversity of ethnicity, language spoken, parity, remoteness from District Hospital (determined at the commune level, range 4–45 km), and degree of health service use (so as to include women who had previously given birth at health facilities and women who had given birth at home). We believe that the variation of experience present in the data is sufficient to adequately support the reported results and answer the research questions (Morse, 1995).

### Data Collection

Focus groups were comprised of between 4 and 8 women and lasted between 43 minutes and 1 hour 53 minutes. We conducted seven focus groups in the homes of community leaders and one in a community hall. Each session was comprised of several sections: introduction and consent process, warm-up discussion, focus group discussion **(Figures A and B)**, and a closing demographic questionnaire. Focus groups were conducted primarily in Vietnamese, with some interpretation into Thai and Hmong, facilitated by a woman Vietnamese researcher with a nursing background (D.T.L.), under supervision of a woman Australian doctoral student with extensive qualitative research experience (S.M.). Interpretation into local languages was performed by local women, including representatives of the VWU, the People's Committee, and a village midwife, who also gave a key informant interview. We audio recorded focus groups and took detailed field notes. One key informant interview was audio recorded; the other was documented by field notes as the participant did not wish to be recorded.

### Data Analysis

An independent third party translated audio recordings and transcribed them verbatim in English. Data were managed using NVivo 11 software (NVivo, 2015). We conducted a thematic analysis, employing an iterative inductive (data-driven) and deductive (researcher-driven) approach (Bradley, Curry, & Devers, 2007). Upon completion of initial coding, themes and relationships between and among codes were identified. These emergent themes were found to be aligned with Edwards' et al. (2015) conceptualization of health literacy as a distributed, shared asset, which provides the broad framework for the presentation of our findings. Although this conceptualization has previously been applied to people living with chronic conditions, due to the alignment of our analysis and the distributed health literacy concept, we feel that the application of the model to the maternal health context is appropriate.

## Results

The results of the thematic analysis are presented under four subsections corresponding to the areas of distributed health literacy (Edwards et al., 2015) as described above. The fourth subsection on supporting decision-making also discusses the subtheme of nonsupportive decision-making, where the influences of social networks and collective decision-making suggested a negative effect on health behaviors.

### Shared Knowledge and Understanding

Shared knowledge and understanding were important elements of distributed health literacy among Thai and Hmong women.

***Experienced women are knowledgeable women.*** Pregnant women and mothers of young children drew on formal and informal sources of information, with emphasis placed on knowledge shared by older, experienced women, especially within the immediate family group. Women who had given birth and raised children were seen as trusted sources of information. Their experiences conferred considerable perceived credibility to their knowledge and advice.

“My experienced mothers – I follow their advice. They have had many children so they must know a lot, so I just follow them.” (Thai, mothers of children under five years focus group [Mu5FG])

Knowledge shared by older women was seen as an inheritance of sorts; with understanding of maternal and child health imparted to younger generations as they went through the experience of pregnancy and becoming a mother, like their mothers before them.

“It is passed down from my mother to me to my daughter.” (Thai, Grandmothers focus group [GFG])

Women who had experienced pregnancy and motherhood were also frequently mentioned as sources of information for women in recognizing signs of pregnancy and encouraging them to take a pregnancy test.

“I felt tired so I asked my mother and my sister. My mother told me to use the test stick and it showed that I'm pregnant.” (Thai, mixed focus group – pregnant women and mothers of children under five [MFG])

“Other women told me that if I don't get my period, I have to buy pregnancy stick. I learned from them.” (Hmong, Mu5FG)

***Traditional knowledge: “that's how Thai people do it.*”** A notable time when shared knowledge came to the fore was during the postpartum period. Thai women talked about their postpartum practices, dictated by tradition and shared knowledge among Thai people. Thai women took herbal baths multiple times a day for approximately 1 month post-partum. The mother or mother-in-law of the postpartum woman generally prepared the baths, as they knew which herbs to use.

“All kinds of herbs. Only old people know. All Thai women do that after they give birth.” (Thai, pregnant women focus group [PWFG])

This practice seemed to be considered a compulsory practice among the Thai participants. However, the practice was adapted to accommodate the fact that women are now more likely to give birth in a facility, compared to their mothers. Thai grandmothers discussed how they implemented and adapted traditional postpartum bathing practices within their families.

“Women who have just had a baby should take a bath 3 times per day. Early morning I would go pick the herbs and make a bath for my daughter. She has to do that for 1 month after giving birth. That's how Thai people do it. Now pregnant women give birth at the hospital and only bathe once a day. But when they come home, we tell them to take herbal bath too. So that they're healthy. And must be herbal baths, not regular baths.” (Thai, GFG. Quotes from multiple participants edited together for clarity.)

### Accessing and Evaluating Information

***Family members as (health) literacy mediators*.** According to Provincial policy, all women in DBP receive a Maternal and Child Health Handbook when they present to a health station for antenatal care; however, not all of the women who participated in the study could read it. Some participants (the majority of the Hmong women and Thai grandmothers) had limited Vietnamese language and reading skills. More educated family members, particularly husbands, were called to act as literacy mediators, to help women access information.

“Her husband read the Handbook and explained to her a bit.” (Hmong, MFG, through local translator)

Other women mentioned reading information in the Handbook themselves, then discussing it with family members, such as their husband and parents.

“She said she often reads the handbook but there are parts that she doesn't understand. Then she asks her husband to read the advice in there.” (Thai, PWFG, through local translator)

***Community members as health literacy mediators*.** For one group of Hmong women in our sample, the village midwife was an important health literacy mediator, providing advice and subsidizing or sometimes filling gaps in communication from health professionals at the health station or hospital, where Hmong women often experienced language barriers. For these women, access to the village midwife meant that they did not need to spend time seeking out and evaluating health information on their own because there was someone in, and from, their community who could give them advice in their language.

“I know it from [name of village midwife]. I receive nothing when I go to the health station or hospital.” (Hmong, Mu5FG)

“I'm just like them and they're just like me. The village health worker is a man and couldn't do this work so I was trained to do this. I live in this village my whole life.” (Hmong, village midwife)

The midwife was able to “find and visit” women opportunistically when she heard about new pregnancies and visited women at night in their homes when their work was done and the women were available to talk.

### Supporting Communication

As described above, social networks were often vital for facilitating the exchange of information between health professionals and ethnic minority women, acting as literacy and health literacy mediators. Participants did not explicitly discuss how their family and social networks supported them with routine direct interactions with health professionals (e.g., attending antenatal care check-ups). However, there were cases where family members, particularly older family members, took on roles advocating for women within the health system, particularly when women's symptoms were dismissed and/or misdiagnosed. One woman described an incident in which her parents and parents-in-law disagreed with health station staff when she was experiencing stomach pain and nausea. Her family was certain she was pregnant, despite a negative home pregnancy test. Health staff at the commune health station were unable to diagnose her pregnancy after a week of inpatient care. Eventually, her father-in-law insisted she receive an ultrasound to confirm the pregnancy.

“I stayed at the health station for 1 week, and they didn't know that I'm pregnant either. They said it's just stomach-ache and nausea. They told me to get the referral to transfer to the district hospital. My parents didn't want me to go to the hospital, they insisted that I was pregnant.” (Thai, MFG)

### Supporting (and Not Supporting) Decision-Making

Family members played an essential role for many women in making decisions about accessing antenatal care and delivery location. Decisions were often made by family members, particularly parents or parents-in-law, with what seemed to be limited input from pregnant women. This collectivist, family-based approach to decision-making resulted in family networks both supporting and undermining women's preferences.

“My family also supported and encouraged me to give birth at the hospital. If I gave birth at home, I wouldn't know anything.” (Thai, Mu5FG)

“One time I wanted to go to the health station for a checkup but my parents said I didn't have time so I shouldn't go. I already had one check-up before so that's enough.” (Hmong, Mu5FG)

Women often said that they would listen to the advice given by health professionals over conflicting advice from their families. However, it appeared that in practice, family members' preferences often prevailed, particularly when making decisions about when to access health services, and/ or if there were logistical obstacles to overcome in accessing services.

***Choosing where to give birth***. When choosing where to give birth, traditional customs seemed to be less influential than family influence. Family influence could both facilitate and prevent women giving birth at a health facility. However, family members' decision-making often seemed to be influenced by circumstances relating to the timing of labor and delivery, availability of safe transportation (typically a motorbike), and financial considerations, rather than a rejection of facility-based births. Indeed, families of pregnant women often stated in-principle support of facility-based births. The participants in the grandmothers' focus group were particularly positive about the improvements in maternal health and services between their time and their daughters' generation.

“In the past we gave birth at home but it was very difficult. Now at the hospital, pregnant women are welcomed and supported by doctors.” (Thai, GFG)

However, in practice, women's and families' preferences regarding delivery location were sometimes influenced by the circumstances outlined above, particularly if the risks and/or costs of travelling to a health facility were perceived by family members to outweigh the benefits. Some women's decisions were strongly influenced by family members, or the decision was taken out of their hands altogether.

“I was afraid that I'd never given birth before. My parents said that my sisters all gave birth easily, my mother too, so it's ok to give birth at home.” (Thai, Mu5FG)

The woman speaking in the following quote went into labor at night, during the rainy season. She wished to deliver in a hospital, but circumstances combined with her mother-in-law's assessment of the risk in travelling to hospital saw her deliver at home instead.

“I still thought I should go to the hospital, but my mother-in-law said the road is too slippery. And my father-in-law was drunk. Many people [advised me to give birth in hospital], but my mother-in-law decided already.” (Thai, Mu5FG)

***Nonsupportive decision-making***. A few cases of what could be referred to as nonsupportive decision-making— where decisions were made that did not align with a woman's preferences and may negatively affect a woman's or a child's health—also arose, reflecting that women sometimes have little power to make decisions about their pregnancy and their children's health. One woman described how she was pressured by her husband to have a son, having already given birth to four daughters (three surviving). At the time of the study, she was pregnant, and planned to go to the provincial capital to find out the sex of her fetus via ultrasound and have an abortion if it was another daughter. She would continue to try to have a son to fulfill her husband's wishes, against the advice of health staff and despite financial strain.

“My husband (…) tells me to have a boy, otherwise there's no one to look after the house. [Health staff] told me to stop but (…) We don't have a boy so I have to keep giving birth (…) We're in such a difficult situation. If we have too many children, we can't support them all.” (Thai, PWFG)

Another woman mentioned that exclusive breast-feeding could be interrupted and weaning commenced earlier than advised if parents-in-law were strict and demanded that a new mother return to work.

“If my parents-in-law are strict and hard to please, I can't keep staying home with my baby for more than 2 or 3 months after giving birth. I have to go to work. I can't breast-feed my baby, then I have to wean.” (Thai, Mu5FG)

## Discussion

These results demonstrate that distributed maternal health literacy is evident in DBP. Ethnic minority women drew upon family and social networks to share knowledge and understanding, assess and evaluate information, communicate with health professionals, and support decision-making. These results are also supported by our previous studies in DBP. An interview study with health professionals demonstrated that they often enlisted women's family and community members to act as interpreters and advised women to ask family for assistance in assessing written health information, placing much of the burden of communication issues and understanding health information on ethnic minority women and their families (McKinn et al., 2017b). We also found that women's social and family networks can contribute to nonsupportive decision-making, that is, decision-making that did not align with women's stated personal health and reproductive preferences, and may lead to negative health outcomes.

Family networks play an important role in both facilitating and, importantly, delaying access to care, particularly during labor. Women in this study described situations in which their decision about where to give birth was influenced or overruled by older family members, particularly when there were extenuating circumstances, such as wet weather or lack of safe transportation. Particularly in the absence of an obstetric complication, this could be perceived as a rational benefit/harm trade-off given that the most readily available transportation for a laboring woman is the back of a motorbike on narrow paths. These factors affect and delay the decision to seek care, as well as potentially delay arrival at a health facility. They correspond with the three phases of delay framework (Thaddeus & Maine, 1994). The framework outlines the three phases of delay between the onset of an obstetric complication and the outcome of maternal death: (1) deciding to seek care, (2) identifying and reaching a medical facility, and (3) receiving adequate and appropriate treatment. The framework was subsequently extended to include preventive care-seeking for an anticipated normal delivery (Gabrysch & Campbell, 2009). There are opportunities in DBP for community educational interventions around the factors influencing first- and second-phase delays to encourage preventive facility delivery, particularly when women want to deliver in a health facility.

Findings regarding family influence over decision-making are not unique to ethnic minority populations in Vietnam. Women's limited autonomy in decision-making should also be reflected on in regard to a woman's position in the family and in Vietnamese society more generally. Although women are advancing in areas such as labor force participation (World Bank, 2017), there is evidence of son preference in an increasing male-to-female sex ratio at birth in Vietnam, affected by increasing access to inexpensive ultrasound technology (den Boer & Hudson, 2017; Guilmoto, 2012). Women in our study spoke openly about finding out the sex of their baby via ultrasound, although prenatal sex identification is illegal. The influence of traditional gender roles is still strong within the domestic sphere, and the needs of the family dominate the needs and rights of the individual (Graner, Mogren, Duong, Krantz, & Klingberd-Allvin, 2010; UNICEF Viet Nam, 2011). Previous research in Vietnam has also found that pregnant women may hold relatively little individual autonomy to make decisions about their health (Graner et al., 2010), particularly in relation to their husband and parents-in-law, as families may collectively take responsibility for decision-making. For this reason, health professionals and community leaders need to ensure that those family members who do make health decisions are involved in discussions about maternal and child health. These findings related to gender roles and equity, as well as the above-mentioned findings regarding the availability of transportation and financial considerations, also suggest the importance of the social determinants of health as factors in distributed and maternal health literacy, which have previously been found to be related to low maternal health literacy scores in the U.S. (Mobley et al., 2014; Smith & Carroll, 2017). This relationship could be an avenue for future research.

Wider peer and community networks are important in improving health literacy in low- and middle-income settings (Batterham, Hawkins, Collins, Buchbinder, & Osborne, 2016). A cohort study in Ghana found that maternal health literacy could be improved through group antenatal care, with women who participated in group care demonstrating an improved ability to understand and operationalize health information over those who received individual antenatal care (Lori, Ofosu-Darkwah, Boyd, Banerjee, & Adanu, 2017). In Vietnam, community-embedded workers such as trained ethnic minority village midwives have been shown to be important resources for ethnic minority women (Doan et al., 2016), which was also supported by our findings. Community and group-based health promotion activities are already common in Vietnam (Kulathungam, 2016; Rheinländer, Samuelsen, Dalsgaard, & Konradsen, 2011; Takanashi et al., 2013), but could be better used to improve the health literacy of the wider community and families, as well as pregnant women and mothers of young children. Previous maternal and child health education activities in DBP have successfully engaged and involved community leaders (Hoc Mai Foundation, 2014), and older women willingly took part in the current study, which suggests that they may be interested in being involved in maternal and child health promotion activities. Further research is needed to fully ascertain the feasibility and acceptability of involving family members and other influencers in health promotion activities and education.

Strengths of this study include a heterogeneous sample, a rigorous analysis process, and the involvement of local collaborators. The main limitation of this study is that Vietnamese is not the first language of the ethnic minority people living in this community. Most women who participated in the study spoke Vietnamese; others needed to speak through local interpreters. The use of local interpreters may have resulted in some distortions in women's responses, either self-imposed or interpreter-imposed. This is a cross-cultural study, and as such, some responses may have been misinterpreted by the authors. We have attempted to limit misinterpretations by conducting an independent translation of audio data and collaborating with a Vietnamese coauthor (D.T.L.). Any actual or potential misunderstandings were discussed by authors in regular meetings during data collection. Additionally, self-reported practice may differ from actual behavior, and there may be a related element of social desirability bias. We have tried to minimize this through use of a neutral facilitator and assuring participants about confidentiality. Finally, as this is a qualitative study, the generalizability of these findings may be limited. However, we have taken several steps to enhance transferability and assist the reader to determine the relevance of these findings to other settings by thoroughly describing the research context and methods (Lincoln & Guba, 1985), and relating our results to existing models and evidence from the literature.

## Conclusion

Family members are an important source of information and advice for pregnant ethnic minority women, and an important influence on decision-making during pregnancy and childbirth. Distributed health literacy can be used both to strengthen health promotion messages, and to reduce the risk of negative health outcomes for women by increasing understanding among key family and community members. Health information is often delivered to ethnic minority women in a way that places the responsibility of understanding and operationalizing information on individual women. In practice, health knowledge and the responsibility for making decisions about pregnancy and childbirth are distributed through women's social and family networks. However, this knowledge may be based on personal experience and cultural traditions rather than medical evidence, which has implications for health outcomes. Older relatives (particularly mothers and mothers-in-law) and husbands of pregnant women could be included in community maternal health education in the future, although further research is needed to ascertain feasibility and acceptability. This may strengthen the health literacy of the community as a whole, and the distributed health literacy resources that ethnic minority women draw upon to maintain their health and the health of their children.

## Figures and Tables

**Table 1:** Participant Characteristics (*N* = 42)

**Characteristic**	**Number**
Pregnant women and mothers of children younger than age 5 years	

Age (years)	
<20	7
20–24	21
25–29	5
30–34	4

Ethnicity	
Thai	28
Hmong	9

Years of school	
None	5
1–6	10
7–12	19
Post high school	3

Number of children	
0	9
1	14
2	12
3	1
4	1
Currently pregnant	16

Grandmothers of children younger than age 5 years	

Age (years)	
45–49	2
50–54	2
55–59	1

Ethnicity	
Thai	5

Years of school	
None	1
1–6	4

Number of children	
2	1
3	1
4	2
5	1

Number of grandchildren	
1	1
2	2
3	0
4	1
5	1

Key informants	

Age (years)	
25–29	2

Ethnicity	
Hmong	2

Years of school	
7–12	2

**Figure A.**
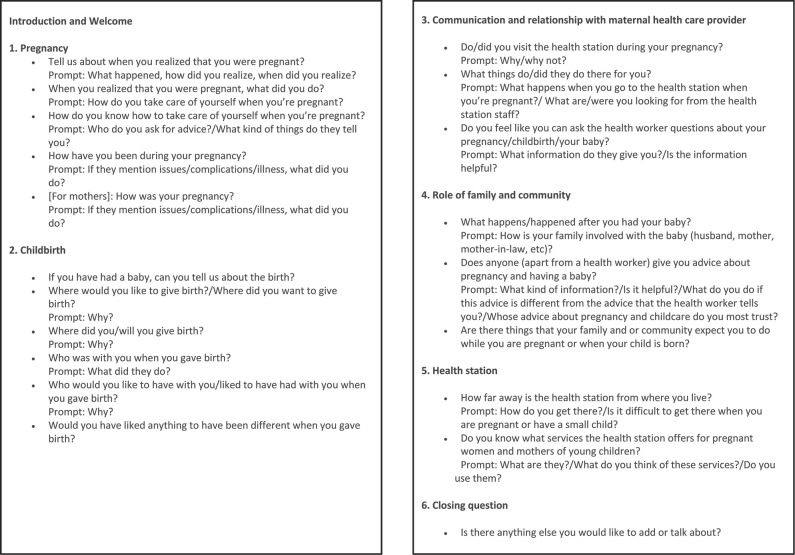
Focus group discussion guide: pregnant women/mothers.

**Figure B.**
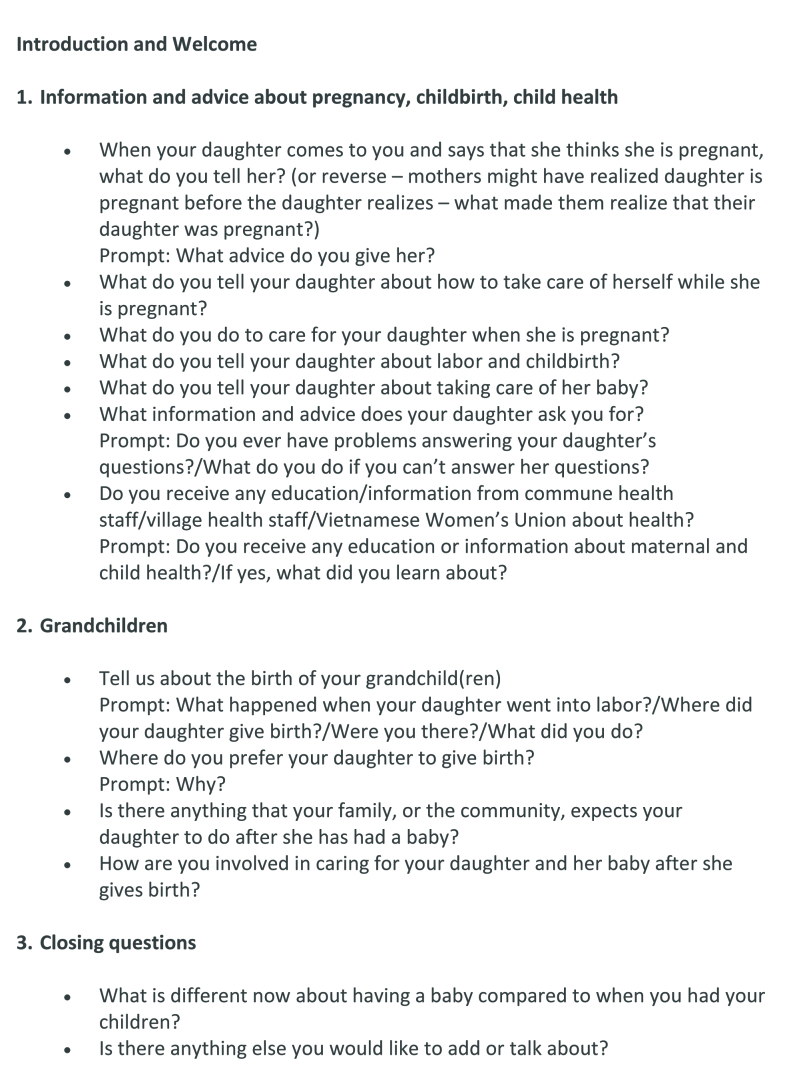
Focus group discussion guide: grandmothers.
